# Supplementary material for: Are carbon emissions trading and green financial instruments synergistic? -Comprehensive quantitative research based on content analysis
Source: PLoS One. 2024 Mar 7;19(3):e0298601. doi: 10.1371/journal.pone.0298601 (PMC10919601; doi:10.1371/journal.pone.0298601)
Supplement: S1 Data — (ZIP) [file pone.0298601.s001.zip › Data/地级市Z_F工作报告.zip!/║■▒▒/╧σ╤⌠╩╨/└┤╫╘╣1⁄2╓┌║┼í╛┬φ┐╦╩2╛▌═°í┐_2018.docx]

各位代表：

　　现在，我代表市政府向大会报告工作，请予审议，并请政协委员和其他列席人员提出意见。

　　一、2017年工作回顾

　　过去的一年，我们在市委坚强领导下，坚决贯彻落实中央和省委省政府决策部署，团结依靠全市人民，以新发展理念引领发展全局，以供给侧结构性改革推进转型升级，“一极两中心”建设迈出坚实步伐。

　　——综合实力显著增强。预计地区生产总值突破4000亿元，居全省第二位，人均地区生产总值突破1万美元，高新区综合实力进至全国国家级高新区第31位，枣阳市连续两年跻身全国百强县（市）行列，全省考核的7个县（市）区均被评为“全省县域经济工作成绩突出单位”。

　　——经济发展质效双升。工业结构优化、增长稳健，预计全年26个行业实现两位数增长，工业用电量增幅和工业品产销率高于全省平均水平，1-11月制造业税收同比增长12.1%。服务业发展加快、效益提升，1-11月第三产业税收同比增长18%，占全口径税收的比重达到47.6%，同比提高3.3个百分点。就业增长超预期，全年城镇新增就业11.2万人，同比增长23%。品牌建设成效显著，成为全国消费品工业“三品”战略示范城市，地理标志商标数量居全省第一，境外世界500强企业新增3家、达到29家，骆驼集团连续五年上榜“中国民营企业500强”。

　　——新的动能加快成长。改革释放新动能，有效激发了创新创业活力，53项国家和省级改革试点落地实施，商事制度改革、推广PPP模式、土地集约节约利用等三项改革获得国务院表彰激励，荣获两个“湖北改革奖”，全年新引进高层次创新创业团队32个，新增市场主体9.4万户。传统产业焕发新活力，预计全年汽车、装备制造、建材冶金产值同比分别增长13%、14.5%、15.5%，汽车产量突破30万辆，创历史最高水平。新产业加速壮大，美洋、九州、雅致等重大项目相继投产，东风新能源汽车产量同比增长80%，全市新能源汽车产量突破2.7万辆，我市获批国家新能源汽车关键零部件创新型产业集群，“中国新能源汽车之都”底气更足。新技术持续突破，获得省级以上科技成果200项，新增高新技术企业65家、省级高新技术产品70个，高新技术产业增加值占地区生产总值比重居全省前列。

　　——城市影响力明显提升。创建全国文明城市形成了广泛共识、凝聚了社会合力，城市面貌焕然一新，市民素质大幅提升，20个单位和家庭分别荣获全国文明单位、文明村镇、文明校园、文明家庭等称号。我市夺得全国综治“长安杯”，被评为首批国家食品安全示范城市，高分通过国家卫生城市复审。举办襄阳马拉松赛和诸葛亮文化旅游节，吸引大批国内外人士集聚襄阳、全国主流媒体瞩目襄阳。国际“朋友圈”进一步扩大，与6个国际城市新建立了友好关系。

　　——民生福祉不断增进。城乡居民人均可支配收入与经济发展同步增长，发展成果惠及更多群众。

　　省定年度脱贫任务全面完成，为贫困家庭学生发放生活费补助、为贫困人口购买商业医疗保险等扶贫举措取得良好效果。对困难群众实施医疗救助10.2万人次，为324371名适龄妇女做了免费“两癌”筛查，为73585名残疾人发放了生活补贴或护理补贴，14000户城镇居民和8150户农村扶贫搬迁群众住上了新房，解决了18.6万农村居民饮水安全问题，城镇职工最低工资标准、城乡低保对象补助标准、城乡特困人员供养标准进一步提高，城乡居民医保住院报销比例全省最高。

　　一年来，我们主要抓了七个方面的工作：

　　（一）抓招商、上项目，投资支撑明显增强。全年招商引资实际投资额突破2400亿元，新引进投资过5亿元的项目218个，其中4个工业项目投资过50亿元、52个投资过10亿元。项目建设快速推进，27个省市重点项目、207个亿元以上工业项目竣工投产，其中猛狮动力电池、东风井关、恩梯恩传动扩能等23个项目投资过10亿元，预计全年固定资产投资、民间投资、工业投资同比分别增长15%、15.2%和22%。

　　（二）抓服务、稳增长，实体经济回暖向好。拿真金白银支持实体经济，全年为企业减税降费57.4亿元，兑现惠企资金超过10亿元。促进银企精准对接缓解融资难，预计全年新增贷款240亿元，贷存比同比提高1个百分点。强化政策措施支持企业引进人才，吸引653名博士硕士落户、2.3万名大学生就业；为751家企业发放稳岗补贴，稳定职工岗位11.9万个。

　　（三）抓调整、优结构，产业升级步伐加快。强力推进工业转型升级，预计全年技改投资同比增长50%，93个亿元以上技改项目、95个亿元以上新兴产业项目竣工投产，6家企业成为全省智能制造试点，风神汽车产值突破330亿元、创历史新高，中航机电成为百亿企业。加快发展现代服务业，金鹰重工公铁联运物流园一期等重大物流项目建成运营，中心城区28个专业市场迁入樊西市场集聚区，实现业态升级；“襄阳云谷”云计算大数据中心服务企业1.8万家，电子商务交易额同比增长25%；古隆中5A级景区创建通过省旅游委验收，唐城游客人次突破百万大关，乡村旅游持续火爆。扎实推进国家现代农业示范区和“中国有机谷”建设，新认证“三品一标”产品155个，建成高标准农田108.1万亩，战胜历史罕见的秋汛和大面积旱涝灾害，粮食总产量稳定在百亿斤以上，枣阳成为全省“优质粮食工程”示范县（市），我市农民合作社和家庭农场总数居全省第一。

　　（四）抓创建、提品质，城乡面貌显著变化。举全市之力开展文明创建，有力推进了市容环境专项整治、违法建筑综合治理、市政设施功能提升，东西轴线、鱼梁洲环岛景观带等35个重点城建项目开工建设，改造棚户区8788套，修补主次干道112条，新建绿道30公里，新增绿地500万平方米，城市功能品质显著提升。加强综合交通枢纽建设，三条铁路和四条高速公路襄阳段建设快速推进，新建一二级公路155公里、农村公路1153公里；注册设立了襄阳航空公司，襄阳机场新航站楼投入使用，旅客吞吐量首次突破百万人次大关；雅口枢纽、新集枢纽、襄阳新港等航运体系建设有序推进。加大环境保护力度，中央环保督察反馈问题整改年度任务全面完成，汉江干流襄阳段水质持续稳定，水库投肥养鱼污染、禁养区养殖污染、黄标车污染、燃煤小锅炉污染等一批突出问题得到有效解决，谷城县“城市矿产”示范基地通过国家验收。积极推进美丽乡村建设，112个乡镇生活污水处理厂（站）全面开工，78个建制镇生活垃圾中转站和83个100立方米以上农村沼气工程全部竣工，宜城市荣膺“国家园林城市”，南漳县成为“全省森林城市”，建成4个省级森林城镇、119个省级绿色示范乡村。

　　（五）抓改革、促开放，内生动力持续增强。持续推进放管服改革，工业项目“零审批”“多证合一”“只跑一次”等改革举措落地见效，行政审批和政务服务“一张网”在全省市州率先建成。积极推进投融资体制改革，汉江国有资本投资集团组建运营，汉江产业基金参股投资重点项目17个、撬动产业资本32亿元，PPP项目撬动民间投资31.7亿元，新增直接融资86.8亿元。扎实推进农村改革，宜城土地制度改革三项国家试点取得积极成效，农村承包土地“三权分置”、集体林权制度和国有林场改革走在全省前列。全力推进襄阳自贸片区建设，保税物流中心等开放平台功能进一步提升，引进国内外产业项目40个、总投资268亿元；融入“一带一路”取得新进展，我市3家企业实施了4个国际产业合作项目；对外贸易质量进一步提升，机电产品出口额居全省第二位。

　　（六）抓保障、兜底线，人民生活持续改善。坚持在发展中保障和改善民生，持续加大民生投入。切实加强社会保障工作，“全民参保登记计划”全面完成，机关事业单位养老保险制度改革并轨全面实施，“五险”扩面新增22万人次；跨省异地就医实现即时结算，城乡居民医保制度整合到位、红利增多；保障农民工工资“三项制度”全面落实。着力提升公共服务水平，新建改建公办幼儿园35所，完成了义务教育“全面改薄”年度任务，高考重点线上线率居全省前列，创造了“七年八状元”的辉煌成绩，湖北文理学院申报专业硕士点通过评审；文化小康村试点工作扎实开展，市规划展览馆正式开馆，图书馆新馆、文化艺术中心、全民健身中心二期工程主体完工；“三医联动”改革在全省考核中名列第一，以药补医机制全面破除，县域内就诊率保持全省领先，计划生育服务管理水平进一步提高。全力推进平安襄阳建设，“三化五联”综治中心规范化建设模式在全国推广，有效化解了一批信访积案，安全生产和食品安全形势平稳，社会持续和谐稳定。

　　（七）抓作风、提效能，政府自身建设加强。以“作风建设年”活动为契机，深入开展效能革命，强化重点工作路线图管理、重点项目拉练和在线督查等机制，推动重点工作月度督查约谈和“市民问政”常态化，有力促进了政府工作效能提升。以开展全省法治政府建设试点为抓手，深入推进依法行政，自觉接受市人大和市政协监督，认真办理人大代表议案、建议和政协建议案、提案，认真对待社会监督和舆论监督；以正风肃纪为重点强化从严治政，推进政府系统“两学一做”学习教育常态化制度化，压实全面从严治党主体责任，严肃党内政治生活，持续加强党风廉政建设，严格执行中央八项规定精神和实施细则，清廉政府建设取得新成效。

　　与此同时，国防动员、人民防空、“双拥”共建、对口支援等工作继续加强，慈善、助残、妇女儿童和红十字人道事业实现新的发展，审计、统计、物价、供销、气象、档案、保密、史志、对台、外事侨务、民族宗教、机构编制、公共资源交易等方面的工作都取得了新的成绩。

　　各位代表！过去的一年，我们在充满希望与艰辛的路上砥砺前行，“一极两中心”建设实现良好开局，每一项发展成果都凝结着全市广大干部群众的智慧和汗水。在这里，我代表市政府，向全市人民，向各位人大代表、政协委员，向各民主党派、各人民团体和各界人士，向驻襄人民解放军、武警官兵和公安干警，向所有在襄投资者、创业者、建设者，致以崇高的敬意！向所有关心支持襄阳发展的中央部委、省直部门、兄弟城市和海内外友好人士，表示衷心的感谢！

　　各位代表！回顾过去的一年，我们也清醒地看到，工作中还存在着许多不足和差距，主要是：经济下行压力较大，实体经济依然困难，地区生产总值等经济指标增速未能达到预期；供给侧结构性矛盾突出，工业交替领先的产业结构尚未形成，现代服务业和现代农业发展滞后；新的动能支撑不足，创新能力不强、体制机制不优、开放水平不高的问题比较突出；环境保护任重道远，十件民生实事中空气优良天数差3天没有完成省定目标，乡镇生活污水处理厂（站）建设没有达到预期进度；发展不平衡不充分，中心城市辐射带动能力不够，棚户区改造等重点城建项目进展不理想，县域经济实力不强，脱贫攻坚任务十分艰巨；政务服务质量和效率与企业和群众的期盼还有较大差距，政府治理体系和治理能力现代化水平有待进一步提高。我们将正视矛盾，直面挑战，以强烈的责任担当、改革创新的举措、扎实过硬的作风，全力解决这些突出问题！

　　二、2018年重点工作

　　2018年是全面贯彻党的十九大精神开局之年，实施“十三五”规划关键之年，决胜全面建成小康社会攻坚之年。做好今年的工作，我们既面临着结构调整阵痛加剧、实体经济困难增多、民生短板问题突出等带来的巨大压力和挑战，也面临着宏观环境持续向好、国家战略叠加覆盖、新的动能加速成长等带来的重大机遇和有利条件，尤其是习近平新时代中国特色社会主义思想鼓舞人心、催人奋进，全市广大干部群众凝心聚力干事业、上下同欲抓发展的气场日益强大。我们必须审时度势、趋利避害，抢抓机遇、乘势而上，全力推动市委十三届五次全会决策部署落地生根，奋力开创新时代“一极两中心”建设新局面。

　　今年政府工作的总体要求是：全面贯彻党的十九大精神，以习近平新时代中国特色社会主义思想为指导，统筹推进“五位一体”总体布局，协调推进“四个全面”战略布局，坚持以人民为中心的发展思想，坚持稳中求进工作总基调，坚持新发展理念，紧扣社会主要矛盾变化，按照高质量发展要求，以供给侧结构性改革推进“三大变革”，以创新驱动引领转型跨越，以减量化增长推动绿色崛起，以共建共享增进民生福祉，积极探索结构优、质量高、效益好、生态美、百姓富的发展新路，全力提升“一极两中心”建设水平，为湖北“建成支点、走在前列”、全面建设社会主义现代化强省作出新的更大贡献！

　　今年的主要预期目标是：地区生产总值增长8%左右，规模以上工业增加值增长8.2%左右，固定资产投资增长11%，社会消费品零售总额增长11%以上，地方一般公共预算收入可比增长8%以上，实际外商直接投资增长9%左右，出口总额增长8%左右，城乡居民收入增长与经济增长同步，全面完成精准脱贫、节能减排、安全生产、计划生育等省定目标。

　　围绕实现上述目标，我们将抓好八个方面的重点工作。

　　（一）加快转换经济增长动力

　　强化投资关键作用。全力抓好招商引资“一号工程”和项目建设“一号任务”。完善产业链招商推进机制，以壮大先进制造业、战略性新兴产业和现代服务业为重点，着力引进“引爆型”“补链型”“环保型”项目，积极承接智能装备制造产业集群转移，引进投资过5亿元的工业项目120个以上，引进世界500强、中国500强及上市公司20家以上，全年招商引资实际投资额突破2700亿元。扎实开展项目拉练，强力推进105个省市重点项目建设，确保竣工投产20个以上；强力推进365个投资过亿元的工业项目建设，确保竣工投产200个以上。

　　强化创新引领作用。深入推进国家创新型城市建设，高质量建设国家可持续发展实验区，支持各县（市、区）创建省级高新区。大力培育创新主体，新增高新技术企业50家以上、创新型试点企业10家以上，新增在孵科技型企业300家以上。鼓励企业加大研发投入，创建国家制造业创新中心。申请发明专利5300件，积极争创国家知识产权示范城市。加快建设华科城、检测认证产业园，吸引研发设计、检测认证等科技服务业态集聚发展。加快推进科技与经济社会融通发展，打通创新成果与产业对接“最后一公里”，转化科技成果100项以上。

　　强化人才支撑作用。深入实施“隆中人才支持计划”，大力开展“我选湖北·智汇襄阳”“院士助襄”“千人计划专家招引”等行动，支持企业引进人才；加强襄阳科技城、大学科技园、优客工场等“双创”载体建设，组建襄阳人才超市服务联盟，建设人力资源服务产业园，积极发展科技金融、天使基金、风险投资，支持高新区创建国家级“双创”区域示范基地；力争全年吸引40个高层次人才团队创新创业，700名高层次人才全职工作，2.1万名大学生创业就业。实施高技能人才培养工程，开展“万名技师培训”行动；全面深化产教融合、校企合作办学模式改革，整体推进现代学徒制改革，积极打造全国重要的先进制造业职业教育基地。

　　（二）加快建设制造强市

　　全力推进产业转型升级。全面推行工业减量化增长模式，争创“中国制造2025”国家级示范区。实施传统产业技改提质工程，推进智能化改造、清洁化生产，新建100条数字化生产线和30个数字化车间、智能工厂、“机器换人”示范企业，全年完成技改投资800亿元以上；扎实抓好际华智能纺织印染生态工业园、三环智能制造产业园、长源朗弘发动机核心零部件智能制造等34个投资过10亿元的产业链关键环节重大项目；支持东风公司加快建设百万辆整车、千亿级产业基地，带动我市汽车产业转型升级，力争今年整车产量突破40万辆，其中新能源汽车产量突破10万辆。推进新兴产业加速壮大，实施亿元以上新兴产业项目117个，重点抓好丰疆高端智能农机、多氟多海立美达新能源商用车等21个投资过10亿元的重大项目，新兴产业投资占工业投资的比重达到30%以上。强力推进军民深度融合，扎实抓好航宇嘉泰、高德红外等11个军民融合产业项目，积极创建国家军民融合创新示范区。全面提升特色产业园区基础设施和创新创业服务功能，规划建设智能装备产业园、新材料新能源产业园、小河临港经济区，推动余家湖工业园转型建设生物医药产业园，推进重点产业集聚集群集约发展。

　　全力培育骨干企业。重点抓好风神汽车、神龙襄阳工厂、骆驼集团等13个年新增销售收入过5亿元的重大增长点，培育10家省级以上行业隐形冠军，新增80家规模以上工业企业；支持金鹰重工、美洋汽车、九州汽车、一飞汽车、猛狮科技、德纳车桥等10家企业做大做强，加快培育百亿级企业。建成现场管理示范企业20家，新增中国驰名商标5件，积极创建全国质量强市示范城市。

　　全力优化企业发展环境。全面落实我市降成本“37条”政策措施，坚决查处和整治涉企乱收费行为，切实降低实体经济成本。实施金融支持技改工程，壮大融资担保机构资本实力，设立续贷“过桥基金”，引导金融机构更好地服务实体经济；坚决打击非法集资等违法违规金融活动，完善不良贷款化解机制，加大对逃废银行债务行为的惩戒力度，切实防范化解金融风险。实行“一业一策”推进产业发展，落实“一企一策”支持优势企业壮大，强化政策措施支持中小企业成长，完善项目服务秘书制，抓实“千名干部进千企”活动，提升服务企业质量和效率。着力构建亲清新型政商关系，坚决兑现惠企承诺，树立“诚信襄阳”形象；深入落实促进民营经济发展“九条措施”，大力支持民营企业发展，认真落实产权保护政策，激发和保护优秀企业家精神，增强企业投资信心。

　　（三）加快发展现代服务业

　　突破性发展数字经济。着力提升“襄阳云谷”区域性云计算大数据中心服务功能，加快实施华为云计算中心扩能和全省政务云灾备中心项目，积极培育大数据交易市场。开展“千企登云”行动，推动互联网、大数据、人工智能与实体经济深度融合；加快建设华为云服务产业园、中国电信“互联网+创新创业”产业园，吸引大数据产业链企业集聚。大力推进政务服务、公共服务、社会治理等领域的“互联网+”应用，打造普惠化智慧城市，带动全市信息化水平整体跃升。

　　突破性发展现代商贸流通业。支持实体零售企业探索“线上+线下”商业模式，积极引进新零售模式；支持大型商超引进国际名店、名企、名品入驻，打造区域性时尚购物之城。提升中心城区专业市场集聚区综合功能，完善农产品批发市场和冷链物流网络，加快建设传化公路港、国际陆港物流园、小河临港物流园等物流园区，着力打造汉江流域商贸物流中心。大力推进电子商务进农村、进社区，积极创建国家和省级电子商务示范基地。

　　突破性发展文化旅游业。坚持“全域旅游”理念，规划建设全域景区，着力打造全国重要的旅游目的地。加快实施华侨城、百里生态丹渠、尧治河矿山公园等27个重点文化旅游项目，力争古隆中成功创建5A级景区，新增两家4A级景区，积极创建湖北旅游名镇、名街、名村，支持保康建设全国生态旅游示范县。加快发展工业旅游、乡村旅游和民宿业。大力发展文化创意产业，支持唐城、汉城壮大影视文化产业，促进“文创”与“文旅”融合发展。加强旅游公路等基础设施建设，争创全国旅游标准化示范城市。强化旅游营销，培育1－2条国内知名旅游线路、10条乡村旅游精品线路。

　　（四）加快实施乡村振兴战略

　　转变农业生产方式。大力推进农业减量化增长，深入开展农业科技“五个一”行动，支持襄州区创建国家农业科技园区，加快建设高质量高效益的国家现代农业示范区。调优种植业结构，新发展专用小麦10万亩、青贮玉米5万亩，新建油茶基地3万亩；编制实施现代茶产业发展规划，推动襄阳高香茶产业做大做强。推广绿色高效种养模式，支持襄大农牧、正大集团、牧原股份建设百万头生猪产业链，支持科尔沁集团建设规模化肉牛养殖基地；新建“稻虾共作”基地10万亩，发展种养游相结合的林下经济基地10万亩。扎实推进“中国有机谷”建设，新增“三品一标”农产品30个，加快建立农产品质量安全追溯体系，打响“生态襄阳、有机厨房”品牌。

　　推进一二三产业融合。加快发展农产品精深加工业，以壮大食品和林产品加工业为重点，力争每个县（市、区）新引进1-2个投资过亿元的行业龙头企业。推进农业与休闲旅游、健康养老、科普教育等深度融合，积极发展“互联网+农业”，鼓励市场主体投资建设田园综合体。健全城乡融合发展体制机制，实施“三乡工程”，推动城市资本、技术、人才与农村资源有效对接。

　　提升农业综合生产能力。全力服务鄂北水资源配置工程，加快建设襄阳分水口配套工程，抓好大中型灌区续建配套与节水改造，完成101座小型水库除险加固，提升防洪抗旱能力。建设高标准农田104.2万亩，推进全面全程机械化示范创建，确保粮食产能稳定在百亿斤以上。

　　深化农村改革。积极推进农村承包土地“三权分置”改革，促进发展适度规模经营。扎实抓好宜城市农村土地制度改革三项试点。总结推广南漳县全国试点经验，全面推进农村集体产权制度改革，促进集体经济发展壮大。深化供销社综合改革，提升综合服务能力。大力培育新型农业经营主体，新增市级以上示范合作社和家庭农场120家。加强农村基层基础工作，健全自治、法治、德治相结合的乡村治理体系，加快培养造就懂农业、爱农村、爱农民的“三农”工作队伍。

　　（五）加快推进城市品质化绿色化集约化发展

　　推进城市空间集约。抓好新一轮城市总体规划编制和“多规合一”，把以人为本、尊重自然、传承历史、绿色低碳等理念融入规划全过程。划定城市开发边界，明确重点开发区域，优化“一心四城”总体布局。以打造精致城市为目标，保护性修复襄阳古城，加快建设庞公新区。以产城融合为导向，加快建设东津新区，提升高新区宜业宜居水平，支持樊城区创建国家级现代商贸物流示范区。以功能对接为重点，推进襄州城区、古隆中风景区与主城区融合发展。以坚守生态绿心为底线，加快建设鱼梁洲城市中央公园。

　　推进城市功能开发。加快旧城有机更新，建立与经济发展水平相适应的棚户区改造征迁补偿机制，妥善做好征迁安置工作，科学规划建设征迁区域。全力打造畅通城市，加快推进东西轴线、外环南线等重点工程，启动环线提速改造工程，着力改造主次干道、打通断头路、完善微路网，优化城市路网结构；积极创建“公交都市”，构建智慧公交系统；规范发展网约车，积极发展共享交通。提升市政设施质量，规划建设海绵城市，加快建设东津新区、庞公新区、樊西功能区综合管廊，全力解决如厕难、买菜难、停车难。

　　推进城市品质提升。开展汉江两岸城市设计，编制城市色彩规划，促进建筑物、街道立面、天际线、色彩和环境协调优美，塑造彰显地域文化特点、体现时代特征、具有滨江特色的城市风貌。大力开展背街小巷、老旧小区、交通秩序等综合整治行动，加快实施汉江风光带、“九水润城”、古城墙亮化等重点生态景观工程，以深化城管体制改革提升精细化管理水平，用三年时间还市民一个更美的襄阳。

　　推进区域融合发展。着力打造全国性综合交通枢纽，全力服务三条铁路建设，加快建设东津高铁站，积极推进襄（阳）常（德）高铁前期工作；全力推进已开工的四条高速公路建设，开工建设襄（阳）南（漳）高速公路，确保老（河口）谷（城）高速公路全线通车，新建改建一二级公路150公里；加快建设襄阳机场综合业务楼和配套工程，积极推进新跑道前期工作，规划建设航空小镇；加快建设襄阳新港、雅口和新集航运枢纽。推进城乡融合发展，实施207国道和217省道改建工程，开工建设河谷大桥、宜城汉江二桥，加强公路危桥和涵洞维修改造，推动中心城区公交线路向县（市）和卫星镇延伸。扎实抓好宜城市、老河口市国家新型城镇化综合试点工作，支持枣阳吴店镇、老河口仙人渡镇、谷城电商小镇等特色小镇建设。

　　（六）加快打造国家生态文明建设示范市

　　强化生态文明建设机制。牢固树立绿水青山就是金山银山的理念，构建“党政同责、一岗双责”的绿色发展责任体系和考核机制，实行最严格的生态环境保护、水资源管理和耕地保护制度，严格落实“河库长制”，探索建立“山长制”“林长制”，完善生态环境监管体制机制，坚决依法制止和惩处破坏生态环境行为。

　　强力推进绿色发展。编制实施汉江生态经济带襄阳沿江发展规划，加快形成以减量化增长模式为主的绿色生产方式。推动高新区和枣阳、宜城、谷城、老河口、襄州等经济开发区循环化改造，确保所有特色产业园区实现环保达标；严把项目准入关，严格落实南漳、保康国家重点生态功能区产业准入负面清单制度；发展绿色金融，培育壮大节能环保、清洁能源等产业，壮大河谷国家新型工业化（再生资源利用）产业示范基地，推进绿色矿山示范区建设，积极开展城市垃圾和农作物秸秆资源化利用，支持谷城县创建国家循环经济示范县。倡导绿色低碳生活方式，深入开展全民节能行动，大力推广新能源汽车，更新新能源公交车200辆，新增绿色建筑200万平方米、装配式建筑40万平方米。

　　坚决打赢污染防治攻坚战。狠抓中央环保督察反馈问题整改，确保按期销号、彻底整改。突出打好蓝天保卫战，重拳治理工业排放、工地和道路扬尘、汽车尾气、燃煤锅炉、餐饮油烟等重点污染源，实现空气质量持续改善。狠抓“一江九河”流域水环境综合治理，全面建成运营省级以上工业园区污水处理厂，基本消灭中心城区黑臭水体，确保市域汉江干流水质稳定在Ⅱ类，打造全国水生态文明示范城市。开展土壤污染防治行动，强化土壤污染管控和修复。实施农村清洁工程，积极推进农村生活垃圾、生活污水和厕所专项整治，加快建设美丽宜居乡村。

　　加快构筑绿色生态屏障。抓好生态保护、永久基本农田、城镇开发边界红线划定工作。深入实施“绿满襄阳”提升行动，推进鄂北生态屏障、汉江生态廊道建设，精准灭荒5.7万亩，绿化库渠180公里，绿化道路1502公里，建成环城林带19公里、环城镇绿带50公里、省界生态防护带16.5公里，创建省级森林城镇3个、绿色示范乡村60个，积极创建西排子河等国家湿地公园、漳河源等国家自然保护区。

　　（七）加快推进重点领域改革和全面开放

　　深化放管服改革。加强相对集中行政许可权改革试点工作，完善审管分离体制机制。实行市场准入负面清单制度，完善工业项目“零审批”制度，推进“多图联审”“多评合一”；深化“互联网+放管服”改革，实施“网上办、马上办、一次办”服务机制改革。推行“多证合一、多证联办”，抓好襄阳自贸片区“证照分离”改革试点。全面推行“双随机一公开”监管方式，加快构建以信用为核心的新型市场监管机制。

　　深化投融资体制改革。完善汉江产业基金运营机制，加强与产业资本和金融资本的合作，股权基金投资企业达到100家以上。大力推进直接融资，力争5家企业在新三板挂牌、200家企业在四板挂牌。鼓励社会资本参与基础设施和公共服务领域建设，组建PPP基金，推进PPP项目资产证券化试点，加快实施50个总投资605亿元的PPP项目。

　　深化财政和国资国企改革。加快建立现代财政制度和预算制度，完善市区两级财政体制，全面实施预算绩效管理；健全地方政府债务管理机制，有效防范化解债务风险。推动国有企业改革，完善现代企业制度，探索建立职业经理人制度，积极发展混合所有制经济。优化国有资本布局，壮大国有资本投资运营集团。

　　构建全面开放新格局。抢抓襄阳自贸片区建设机遇，对接汉新欧等国际物流大通道，加快融入“一带一路”建设。以制度创新为核心，高质量落实国家和省部署的自贸区试验任务；加快转变政府职能，营造国际化、法治化、便利化营商环境；加快建设“四港一区”、国际贸易“单一窗口”、跨境电商公共服务等开放平台，积极申报综合保税区和一类航空口岸，推进通关一体化建设；支持融资租赁公司、商业保理公司等壮大发展，积极培育金融新业态；探索实行共享发展机制，吸引国内外高端产业集聚。推进外贸出口基地转型升级，加快壮大外贸出口骨干企业，积极培育外贸综合服务等新业态。支持优势企业“走出去”，利用国际市场和资源做大做强。

　　（八）加快增进民生福祉

　　优先发展教育事业。加快发展学前教育，全面提升义务教育均衡发展水平，切实解决中小学生课业负担重、“大班额”等突出问题。启动普及高中阶段教育攻坚计划，加快建设襄阳四中新校区，确保襄阳三中新校区和三十六中改建工程投入使用，提高县（市）普通高中和职业高中教育质量和水平。加快发展高等教育，支持湖北文理学院新校区建设，支持襄阳职业技术学院实施二期工程，支持襄阳汽车职业技术学院建设优质高等职业技术院校。

　　提高就业质量和社会保障水平。落实积极就业政策，城镇新增就业7.5万人。全面实施“全民参保计划”，推广养老保险“助保贷”，“五险”扩面新增16万人次；完善城乡居民医保制度，鼓励发展补充医疗保险、商业健康保险。完善社会救助标准动态调整机制，实现困难群体应保尽保、应救尽救。维护妇女儿童、老年人、残疾人合法权益。加大保障性住房建设力度，推动租赁住房建设，加快建立多主体供给、多渠道保障、租购并举的住房制度，促进房地产市场平稳健康发展。

　　坚决打赢脱贫攻坚战。聚焦深度贫困精准发力，全面落实扶贫政策措施，激发贫困人口内生动力，创新产业扶贫举措，加大金融扶贫力度，支持贫困村发展规范运行的专业合作社，帮助有劳动力的建档立卡贫困户实施长、中、短相结合的产业增收项目，推动脱真贫、真脱贫，全面完成精准脱贫年度任务。

　　加快建设健康城市。全域深化“三医联动”改革，做实家庭医生签约服务，推动优质医疗资源下沉，加强基层卫生服务体系建设，继续解决“看病难、看病贵”问题。加强重大疾病防控，抓好贫困妇女“两癌”筛查救治工作，有效防范和处置突发公共卫生事件。倡导健康文明生活方式，广泛开展健康社区、健康单位、健康家庭等创建活动。认真落实全面两孩政策，促进人口均衡发展。鼓励社会资本进入医疗、养老领域，发展大健康产业。

　　加快建设文明城市。按照“一年打牢基础、两年攻坚克难、三年力争实现”的要求，全域实施全国文明城市创建三年行动计划。大力发展公共文化事业，积极推进文化小康建设，完善城乡公共文化设施；广泛开展文体惠民活动，继续办好诸葛亮文化旅游节、襄阳马拉松赛、端午龙舟赛、中国大学生方程式汽车系列赛等重大赛事；实施文艺精品创作生产工程，鼓励创作一批有影响力的精品力作；加强文化遗产保护利用，推进“万里茶道”申遗工作。

　　加强和创新社会治理。巩固全国综治“长安杯”创建成果，加强四级综治中心规范化建设，健全立体化信息化社会治安防控体系和矛盾纠纷多元化解机制，完善社会力量参与平安建设机制，广泛深入开展基层平安创建活动，打造平安襄阳建设升级版。高度重视和认真做好信访工作，积极回应群众关切，及时化解社会矛盾。加强公共法律服务体系建设，促进社会治理法治化。建立“互联网+食品药品安全监管”体系，全域推进省级食品安全示范县（市）和农产品质量安全县（市）创建工作。健全安全生产责任体系和监管体系，扎实开展打非治违专项行动，确保安全生产形势稳定向好。完善应急救援体系，加强综合防灾减灾能力建设。切实加强国防动员和“双拥”共建工作，做好民族宗教、对口支援等工作。

　　在做好上述工作的同时，我们将认真办好以下民生实事。

　　（1）实施脱贫攻坚工程，全年减少贫困人口47257人，出列贫困村58个。

　　（2）实施便民出行工程，中心城区打通江山南路等断头路20条，增设人行天桥16处，新建改建停车位3.6万个，建成智慧停车管理平台；改造城市公交专用道6条，建设公交站（场）7个、港湾式站台50个，开通和调整公交线路5条以上。

　　（3）实施城市人居环境改善工程，全市改造棚户区34406套，中心城区改造20000套、开工建设棚改安置房10000套。中心城区完成160条背街小巷和12个积水渍涝点综合整治；新建公园3个，建设环岘山绿道、樊城环形绿道，新增绿地200万平方米以上。

　　（4）实施便民菜市场建设工程，新建改造中心城区标准化菜市场30个。

　　（5）实施城区供暖工程，建成供暖主管网30公里，集中供暖覆盖建筑面积达到50万平方米以上。

　　（6）实施城乡清洁工程，大力开展“厕所革命”，新建改建61座中心城区公共厕所、50座景点景区旅游厕所，支持各地抓好城镇公共厕所和农村卫生厕所改造；大力开展垃圾治理行动，在中心城区启动实施垃圾分类管理，新建改建10座生活垃圾转运站，在农村全力推进生活垃圾集中收集处置；大力开展污水治理行动，全市所有建制镇污水处理厂（站）建成运行。

　　（7）实施教育惠民工程，全市新建改建扩建公益普惠性幼儿园30所，新增学位2000个；完成义务教育学校“全面改薄”工作，新建改建校舍20万平方米、运动场55万平方米。

　　（8）实施健康养老惠民工程，新建改建7家社区卫生服务中心、12家乡镇卫生院；开工建设养老机构8家，新增各类养老床位2000张。

　　（9）实施文化惠民工程，全年送戏下乡1000场、“欢乐襄阳”文化惠民演出150场、农村公益电影放映25000场，确保全民健身中心二期工程建成投入使用。

　　（10）实施农村基础设施提升工程，新建1000公里农村公路，解决26万人饮水安全问题。

　　三、加强政府自身建设

　　以作风建设“落实年”为契机，持续开展效能革命，全力打造高效政府，以永不懈怠的精神状态、一往无前的奋斗姿态，展现新气象、创造新业绩。

　　坚持旗帜鲜明讲政治。切实加强市政府党组自身建设，牢固树立“四个意识”，坚定维护以习近平同志为核心的党中央权威和集中统一领导，全面贯彻党的基本理论、基本路线、基本方略，坚决落实中央、省委省政府和市委决策部署；严肃党内政治生活，严守党的政治纪律和政治规矩，切实增强对党忠诚、为党分忧、为党尽职、为民造福的政治担当，凝聚推进“一极两中心”建设的强大合力。

　　坚持牢记使命勇担当。建设“一极两中心”是充满挑战的新征程，我们要汲取“红船精神”的磅礴力量，勇于挑最重的担子，敢于啃最硬的骨头，以敢为人先的首创精神扭住发展不放松、真刀真枪抓改革，以百折不挠的奋斗精神攻坚克难不退步、勇往直前不回头，以忠诚为民的奉献精神扎根群众不动摇、为民造福不懈怠，不断开创“一极两中心”建设新局面。大兴学习之风、调研之风，推动各级公务人员在学习实践中培养专业能力，增强担当的真本领、练就担当的宽肩膀。为敢于担当的干部撑腰鼓劲，营造干事创业的良好环境。

　　坚持务实重行抓发展。向形式主义、官僚主义新表现宣战，以解决表态多调门高、行动少落实差等突出问题为重点，全面推行“一线工作法”，推动各级政府和部门“一把手”沉入基层察实情、扑下身子抓落实；全面实行重点工作路线图管理、全过程督查、全媒体晾晒、全方位问责，推动各级干部主动担当履责抓落实；全面实行项目建设月调度、季拉练、年评比的竞赛机制，推动各地比学赶超抓落实；全面建设诚信政府，定下的事说干就干，认准的事一抓到底，承诺的事坚决兑现，把襄阳发展每一步迈稳踏实。

　　坚持从严治政树清风。深入推进法治政府建设，完善依法科学民主决策机制，坚持严格规范公正文明执法，加强行政复议和行政应诉工作，认真实施各项法律法规和政府规章，推动各级政府依法全面履行职责。自觉接受市人大和市政协监督，积极回应社会监督和舆论监督，让权力在阳光下运行。压实政府系统全面从严治党主体责任，扎实推进“两学一做”学习教育常态化制度化，认真开展“不忘初心、牢记使命”主题教育，促进各级公务人员用习近平新时代中国特色社会主义思想武装头脑；持之以恒正风肃纪，严格落实中央八项规定及实施细则精神和省委、市委实施办法，进一步加强办公用房和公务用车管理，严防“四风”问题反弹；坚决支持政治巡察和纪检监察工作，加强对权力运行的制约和监督，严肃查处侵害群众利益的不正之风和腐败问题，着力打造清廉政府。

　　各位代表！新时代呼唤新担当，新征程要有新作为。让我们紧密团结在以习近平同志为核心的党中央周围，在省委省政府和市委的坚强领导下，锐意进取、埋头苦干，为开创“一极两中心”建设新局面而努力奋斗！
